# Chylous and Chyliform Effusions

**Published:** 1906-12-29

**Authors:** J. M. Fortescue-Brickdale


### CHYLOUS AND CHYLIFORM EFFUSIONS.

By J. M. FORTESCUE-BRICKDALE, M.A., M.D.Oxon.

Such a striking phenomenon as the appearance of milk or milk-like fluid in the peritoneal or pleural cavity could hardly escape the least careful observer of pathological conditions, and thus it is not remarkable that "chylous ascites" has long been known; a case is on record which occurred in 1633. It is only comparatively recently, however, that improved methods of examination have shown that these cases include two broad groups, differing in their pathology and in the nature of the fluid effused. The first group is that known as chylous ascites; it contains cases where the fluid found is really chyle which has transuded through the lymphatic channels. The second group is called chyliform ascites. The milkiness of the fluid in cases of this kind is due to the presence of endothelial cells in various stages of degeneration. Both these forms of fluid may be present, in which case the ascites must be designated as mixed chylous and chyliform. Very various reasons have been found to account for the milk-like fluid, and somewhat elaborate classifications have been drawn up. The presence of a milky fluid in any serous cavity can, of course, only be determined by exploration. The specimen for diagnostic purposes should then be submitted to a very thorough chemical and physical examination. In cases of pure chylous effusion the fluid will be found, under the microscope, to contain a large proportion of fatty globules, and will at once become clear on the addition of caustic potash and ether. The proportion of fat in the fluid has been shown in some cases to vary directly with the amount of fat in the diet (Strauss). In the analogous condition of chyluria, special fatty bodies, such as olive oil coloured with Soudan III., brucin, lipanin, etc., have been found in the urine after administration by the mouth, but not after subcutaneous injection. It has also been found that the fat disappears during starvation. Such a condition must be due to obstruction somewhere between the absorbent lacteals and the termination of the thoracic duct in the left innominate vein.

According to Pagenstecher, simple ligature of the left thoracic duct is not sufficient to produce chylous ascites; it is necessary that the finer branches should be obliterated in order to cut off collateral channels. External pressure appears to be the commonest cause, usually by new growths or enlarged glands, or by peritonitic conditions, either inflammatory or carcinomatous. Occlusion of the left subclavian vein is also a frequent cause. Other conditions, such as calculus in the thoracic duct, are rare.

Chyliform effusions, on the other hand, may contain fat, but this will be due to degenerative cell processes. Microscopically, the cellular elements in the fluid will be much more numerous, and will be found undergoing fatty change. It is obvious, however, that if these changes are very advanced, the diagnosis may be by no means easy, as only disintegration products may be present. In other cases, fatty matter may be conspicuously absent; the milky appearance may be due to globulius lecithins and other products of cellular disintegration. In a case recently described by Hutchison a proteid yielding nuclein or paranuclein on digestion was found. It was thought by Senator that these fluids could be distinguished from true chylous fluids by the absence of dextrose, which is constantly found in the chylous transudates; but further investigations have shown that small amounts of reducing sugar may be found in chyliform fluids. The conditions under which chyliform ascites is produced are those in which there is a considerable destruction of the cells lining the peritoneal cavity, such as tuberculosis and malignant growth; cirrhosis of the liver and a special fatty degeneration of the endothelial cells, apparently primary, have also been observed. Carcinoma may give rise to a mixed form, partly by pressure on the ducts, and partly by changes in the peritoneum.

The cases, on the whole, appear to be not excessively rare, and further investigation will probably show many additions to the list. The important thing from a diagnostic point of view is an adequate examination of the milky fluid, without which no case can be properly classified. Simple venous stasis, for instance, may give rise to either form, so that no merely anatomical observations, either post-mortem or at an operation, are sufficient to determine the true ætiology of any given instance.

